# Optical Properties of Secondary Organic Aerosol Produced by Photooxidation of Naphthalene under NOx Condition

**DOI:** 10.1021/acs.est.1c07328

**Published:** 2022-04-06

**Authors:** Quanfu He, Chunlin Li, Kyla Siemens, Ana C. Morales, Anusha Priyadarshani Silva Hettiyadura, Alexander Laskin, Yinon Rudich

**Affiliations:** † Department of Earth and Planetary Sciences, Weizmann Institute of Science, Rehovot 76100, Israel; ^‡^Department of Chemistry, ^§^Department of Earth, Atmospheric, and Planetary Sciences, Purdue University, West Lafayette, Indiana 47907, United States

**Keywords:** optical properties, secondary organic aerosol, NOx effect during photooxidation, atmospheric aging, chromophore characterization, chemical composition

## Abstract

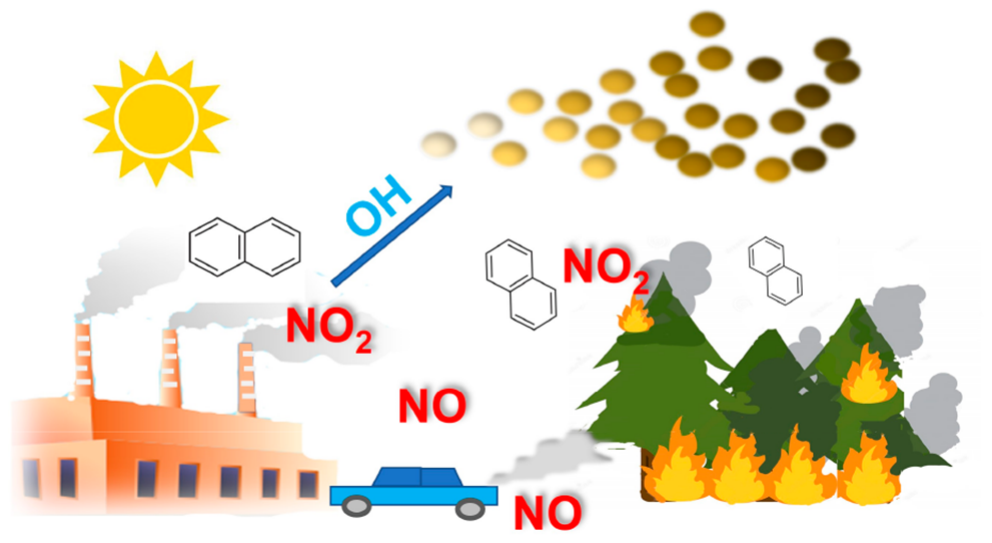

Secondary organic aerosols (SOAs) affect incoming solar radiation by interacting with light at ultraviolet and visible wavelength ranges. However, the relationship between the chemical composition and optical properties of SOA is still not well understood. In this study, the complex refractive index (RI) of SOA produced from OH oxidation of naphthalene in the presence of nitrogen oxides (NOx) was retrieved online in the wavelength range of 315–650 nm and the bulk chemical composition of the SOA was characterized by an online high-resolution time-of-flight mass spectrometer. In addition, the molecular-level composition of brown carbon chromophores was determined using high-performance liquid chromatography coupled to a photodiode array detector and a high-resolution mass spectrometer. The real part of the RI of the SOA increases with both the NOx/naphthalene ratio and aging time, likely due to the increased mean polarizability and decreased molecular weight due to fragmentation. Highly absorbing nitroaromatics (e.g., C_6_H_5_NO_4_, C_7_H_7_NO_4_, C_7_H_5_NO_5_, C_8_H_5_NO_5_) produced under higher NOx conditions contribute significantly to the light absorption of the SOA. The imaginary part of the RI linearly increases with the NOx/VOCs ratio due to the formation of nitroaromatic compounds. As a function of aging, the imaginary RI increases with the O/C ratio (slope = 0.024), mainly attributed to the achieved higher NOx/VOCs ratio, which favors the formation of light-absorbing nitroaromatics. The light-absorbing enhancement is not as significant with extensive aging as it is under a lower aging time due to the opening of aromatic rings by reactions.

## Abstract 2

This study investigates the chemical−physical properties of anthropogenic secondary organic aerosols and the daytime atmospheric transformations of their environmental properties.

## Introduction

Atmospheric aerosols play a critical role in Earth’s radiative energy balance by either warming or cooling the atmosphere through absorption and scattering of incoming solar radiation and outgoing terrestrial radiation. The aerosol radiative forcings of aerosol–radiation interaction and aerosol–cloud interactions are estimated to be −0.22 (−0.47 to −0.04) Wm^–2^ and −0.84 (−1.45 to −0.25) Wm^–2^, respectively.^1^ On a regional scale, the forcing may be much larger. Large uncertainties in radiative and climate forcing are mostly due to organic aerosols, and their light absorption properties are not well understood. Atmospheric brown carbon (BrC) aerosols, which absorb light mostly in the near-ultraviolet to the ultraviolet region, have been recognized to play a critical role in climate forcing, accounting for ∼20% of the absorption by carbonaceous aerosols.^2−6^ BrC is composed of light-absorbing organic compounds with a complex chemical composition and diverse wavelength-dependent light-absorbing properties.^4,7^ The major source of primary BrC is commonly attributed to biomass burning, which is typically treated as the only source of primary BrC in modeling studies.^8−10^ However, multiple field observations^11−13^ and laboratory studies^4,14−19^ demonstrated that BrC can be produced by secondary processes *via* photooxidation and dark reactions^20−22^ of volatile organic compounds (VOCs) from various sources.^4^ Due to the complexity of the chemical composition and their variable wavelength-dependent light-absorbing properties, the investigation of secondary BrC is an active area of research.

Polycyclic aromatic hydrocarbons (PAHs), formed in pyrolytic reactions during fuel-rich combustion of fossil and biomass fuels, are an important class of semivolatile anthropogenic emissions. Light-weight PAHs (<four aromatic rings) exist predominantly in the gas phase and can react with the OH radical to yield oxygenated and nitrated products, which contribute to secondary organic aerosol (SOA) formation.^23−26^ Among the PAHs, naphthalene is one of the most abundant gas-phase species. In the urban atmosphere, typical gaseous naphthalene levels range from 0.01 to 4.85 μg m^–3^ due to high emissions.^24,27−31^ Chamber experiments have confirmed that naphthalene can produce SOA (*naph*-SOA) effectively with SOA yields ranging from 0.03 to 0.96 under different NOx levels.^23,24^ Given the abundant naphthalene in the ambient air and the high SOA yield, *naph*-SOA is one of the major SOA sources in urban areas and downwind as found by both model simulations^32^ and ambient observations.^28,33^ However, studies on the optical properties of *naph*-SOA are rare,^34−38^ and there are no broadband direct online particle optical property measurements that are useful in the estimation of climate effects. The OH oxidation of naphthalene in the presence of nitrogen oxides (NOx) under unrealistic dry conditions produced light-absorbing *naph*-SOA, quantified by measuring the solvent-extracted fraction of *naph*-SOA.^34,35,37,38^ While the study by Xie et al.^35^ identified three species (C_10_H_7_NO_3_, C_10_H_7_NO_4_, C_8_H_9_NO_5_) that account for ∼20% of the measured absorbance at 365 nm using surrogate standards, the other three studies did not provide molecular-level information that explains the observed optical properties of *naph*-SOA.^34,36^ Therefore, comprehensive studies combining molecular-level chemical composition analysis and online wavelength-dependent optical properties for *naph*-SOA are still essential for a predictive understanding of the radiative effect of *naph-*SOA.

Since naphthalene is predominantly emitted from combustion-related sources,^30^ NOx coexists with naphthalene in the ambient atmosphere. Previous studies on the photooxidation of naphthalene have found that the yield of nitro-organic compounds in *naph*-SOA changed with the naphthalene/NOx ratio.^39,40^ However, the influence of the naphthalene/NOx ratio on the optical properties of *naph*-SOA has not been investigated. Previous studies have found that the optical properties (both absorption and scattering) of freshly produced SOAs from both biogenic and anthropogenic VOCs change during the aging process.^14,16,36,41^ These previous studies mainly focused on the photochemical aging process in the absence of NOx. However, in the atmosphere, freshly produced *naph*-SOA undergoes photochemical aging in the presence of NOx during the daytime. Therefore, the evolution of chemical and optical properties of *naph*-SOA in the presence of NOx during aging has to be evaluated to determine the climate effect of *naph*-SOA more accurately.

In this study, *naph*-SOA are produced under atmospherically relevant relative humidity either at the same aging time but with different NOx/naphthalene ratios or under varying aging times at a constant NOx/naphthalene ratio to address the effects of NOx and atmospheric aging thoroughly. *Naph*-SOA is chemically characterized online by a high-resolution (HR) time-of-flight (ToF) aerosol mass spectrometer (AMS). Simultaneously, the size-dependent absorption and extinction cross sections of *naph*-SOA are measured using an optical system consisting of a broadband cavity-enhanced spectrometer (BBCES, 315–345 and 380–650 nm), a photoacoustic absorption spectrometer (PAS, 404 nm), and a cavity-ring-down spectrometer (CRDs, 404 nm). The chemical composition and molecular-specific light-absorbing properties of *naph*-SOA are further assessed offline utilizing a high-performance liquid chromatography (HPLC) separation coupled to a photodiode array (PDA) detector for UV–vis absorption measurement and high-resolution mass spectrometry (HRMS) for molecular characterization. Hence, this study comprehensively investigated the chemical composition, molecular-specific light absorption properties, and online aerosol optical properties of *naph*-SOA produced by the OH-initiated oxidation and the first time to quantitatively assess the impact of the NOx/naphthalene ratio and aging time, which provides experimental insights into the effect of anthropogenic SOA on climate forcing.

## Experimental Methods

### SOA Generation by the OFR

Naphthalene (Sigma-Aldrich) was used as a proxy for anthropogenic SOA precursors. *Naph*-SOA particles were generated by homogeneous nucleation and condensation of oxidized products from OH oxidation of naphthalene in the potential aerosol mass (PAM) oxidation flow reactor (OFR) in the absence of seed particles. The details of the PAM reactor have been described previously.^41−43^ A total flow of 4.3 L min^–1^ of a N_2_ + N_2_O mixture and a 0.2 L min^–1^ O_2_ + O_3_ mixture with a final RH of 36–39% was used, with a corresponding residence time of 184 s. The initial SOA conditions for *naph*-SOA production are listed in the Supporting Information (Table S1), including the initial naphthalene and O_3_ concentrations, RH, and measured NOx concentrations. The NOx/naphthalene ratios varied between 0 and 3.2, as shown in the Supporting Information (Table S1), which are within the typical range of NOx/VOC ratios in the ambient conditions.^44^

OH radicals were produced through the reaction of O(^1^D) with water, whereas O(^1^D) radicals were formed from the photolysis of O_3_ by UV light (λ = 254 nm). N_2_O (99.999%) was introduced to the PAM reactor as the source of NO and NO_2_ generated by the reactions of O(^1^D) + N_2_O → 2NO, and NO + O_3_ → NO_2_ + O_2_.^45,46^ This method provides a more homogeneous NOx environment and can achieve a much higher NO concentration in the reactor than adding NOx directly. The OH concentration in the PAM was controlled by adjusting the UV light intensity. The OH exposures in this study were within the range of (1.04–5.05) × 10^11^ molecules cm^–3^ s, determined by measuring the decay of coinjected SO_2_ (Thermo Fisher Scientific, Model 43i). By assuming a daily average radical concentration of [OH] = 1.5 × 10^6^ molecules cm^–3^,^47^ the equivalent aging time in this study ranged from 0.8 to 4.9 days. Due to the fast conversion of NO to NO_2_ in the sampling line by the high O_3_ concentration, we cannot measure the NO concentration directly. Therefore, only the total NOx concentration after the PAM reactor is recorded (SERINUS 40, Ecotech, Australia).

To investigate the influence of NOx and aging on the optical properties of *naph*-SOA, two sets of experiments were conducted. Five experiments (Table 1, N00–N40) were performed under the same aging time (3.2 days for N00–N20, 2.4 days for N40, which was limited by the reactor), but with different N_2_O inputs (0–4%), to achieve different NOx/naphthalene ratios to test the influence of NOx. Another six experiments (Table 1, A08–A49) were carried out with 2.0% N_2_O, but with different aging times (0.8–4.9 days). This set of experiments was designed to probe the combined effect of NOx and aging time.

**Table 1:** Initial Conditions and Physical–Chemical Properties of *naph*-SOA Generation by PAM

experiment ID	naphthalene	N_2_O	NOx	O_3_	RH	aging time	H/C	O/C	*f*_NO_3__	density
	(ppbv)	(%)	(ppbv)	(ppm)	(%)	days				(g cm^–3^)
A08	345	2	109	48.3	38.8	0.8	1.03	0.67	0.05	1.34
A14	345	2	124	48.3	38.9	1.4	1.03	0.68	0.05	1.32
A22	345	2	188	48.3	38.9	2.2	1.04	0.74	0.06	1.32
A37	345	2	303	48.3	38.8	3.7	1.08	0.86	0.06	1.34
A39	345	2	511	48.3	39.0	3.9	1.09	0.88	0.07	1.35
A49	345	2	1111	48.3	39.0	4.9	1.12	0.92	0.08	1.33
N00	493	0	0	38.4	38.3	3.2	1.05	0.94	0.03	1.37
N05	493	0.5	85	38.4	38.3	3.2	1.06	0.91	0.04	1.34
N10	493	1	209	38.4	38.5	3.2	1.08	0.96	0.05	1.34
N20	493	2	592	38.4	38.2	3.2	1.10	0.96	0.07	1.35
N40	493	4	1352	38.4	38.4	2.4	1.16	0.72	0.13	1.36

The Aerodyne photochemical model implemented in MATLAB (Mathworks) was used to simulate the photochemical reactions in the PAM reactor.^45^ Due to limited knowledge of the reaction mechanism and reaction rate constants of OH oxidation of naphthalene, only a simplified RO_2_ chemical is included in the model, as described previously.^45^ By considering the reaction rate constants of naphthalene with OH, O_3_, and NO_3_, we estimated that more than 98% of the naphthalene was consumed by OH radicals in all of the experiments. More details about the box model are provided in Text S1, and the model output results are shown in Table S1.

### Online Measurement of SOA Chemical Composition and Density

The size distributions of SOAs were monitored by a scanning mobility particle sizer (SMPS, TSI Incorporated, classifier model 3080, CPC model 3775). The chemical composition of *naph*-SOA was characterized by a high-resolution time-of-flight aerosol mass spectrometer (HR-ToF-AMS, Aerodyne Research Inc., Billerica, MA, more details in Text S1), which was operated alternatively in V and W modes. More details about the data processing and instrument calibration can be found in the Supporting Information (Text S1). The effective density (ρ_eff_) of *naph*-SOA particles was derived from measurement of the aerodynamic diameter (*d*_va_) obtained by an aerodynamic aerosol classifier (AAC, Cambustion) and the mode mobility diameter (*d*_m_), which was measured by the SMPS after the AAC.^48^

### Light Extinction and Absorption Measurement

The aerosol extinction cross section, σ_ext_(λ, *D*_p_, *m*), which depends on the light wavelength (λ), the particle mode diameter (*D*_p_), and the complex refractive index (RI, *m* = *n* + *ki*, where *n* and *k* are the real part and imaginary part, respectively) of the material, is described by the expression shown in eq 1
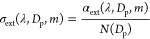
1where α_ext_ is the extinction coefficient of the measured particles and *N*(*D*_p_) is the particle number concentration (cm^–3^). Size-dependent absorption and extinction cross sections are measured using a photoacoustic absorption spectrometer (PAS), a cavity-ring-down spectrometer (CRDS), and a broadband cavity-enhanced spectrometer, which consists of two channels (BBCES_UV_, 315–345 nm; and BBCES_vis_, 380–650 nm). Briefly, aerosols from the OFR pass through an ozone scrubber and then are dried with a diffusion dryer (RH < 20%). The dried aerosols are then passed through a denuder filled with activated charcoal to remove NOx in the gas phase. The outcome particles are size-selected (175–325 nm with 25 nm step) with an AAC and then directed into our optical system for optical property characterization. Meanwhile, the particle number concentration and the mobility size distribution are measured by a parallel CPC and an SMPS, respectively. The experimental setup is shown in the SI (Figure S1), and the operational details of the instruments can be found in our previous studies.^21,41,49,50^

By assuming homogeneous chemical composition of the particles across the different sizes, the complex RI of the aerosols was retrieved by fitting a theoretical Mie curve to the size-dependent extinction cross-section data at each specific wavelength.^51−54^ The retrieval algorithm was limited to searching for *n* ≥ 1 and *k* ≥ 0.

### Offline Characterization of *naph*-SOA Using the HPLC-PDA-HRMS Platform

All details for the offline characterization of naph-SOA using HPLC-PDA-HRMS have been published elsewhere by Siemens et al.^55^ Briefly, *naph*-SOA was collected on Teflon filters (poly(tetrafluoroethylene) PTFE, 0.45 μm pore size, 47 mm diameter, Whatman) for offline analysis. The filters were extracted with acetonitrile (Optima LC/MS grade, Fisher Chemical) under sonication and then analyzed using a high-performance liquid chromatography (HPLC) system coupled with a photodiode array detector (PDA), both from Thermo Vanquish, and a high-resolution (HR) Q-Exactive HF-X hybrid quadrupole Orbitrap mass spectrometer equipped with an electrospray ionization (ESI) source (HPLC-PDA-ESI/HRMS, Thermo Scientific, Inc). About ∼3 μg of dissolved organic matter (OM) was injected into the HPLC for each sample. Analytes were separated on a reversed-phase column (Luna C18, 150 × 2 mm, 5 μm particles, 100 Å pores, Phenomenex, Inc.) using a 200 μL min^–1^ binary solvent mixture containing water (A) and acetonitrile (B), with both solvents containing 0.1% (v/v) formic acid. A 90 min LC gradient was programed as follows: 0–3 min at 10% of B, 3–63 min at a linear gradient to 100% of B, 63–70 min B held at 100%, 70–71 min decreased to 10% of B, and 71–90 min held at 10% of B to re-equilibrate the column. UV–vis absorption spectra were recorded using the PDA detector over the wavelength range of 200–680 nm. Mass spectra were acquired for the *m*/*z* range of 80–1200 Da at a mass resolution of *m*/Δ*m* = 240 000 (at *m*/*z* 200). The raw data were acquired using Xcalibur software (Thermo Scientific) and were further processed using MZmine-2.38.^56−58^

## Results and Discussion

### Online Chemical–Physical Characterization of *naph*-SOA

Despite the changes in the NOx/naphthalene ratio and aging time, the particle effective density did not change significantly (1.32–1.37 g cm^–3^). The particle size distributions of *naph*-SOA are shown in Figure S2. For experiments conducted to study the NOx addition effect (N00–N40), the particle mode diameter of *naph*-SOA was about 97 nm. It decreased to ∼75 nm upon NOx addition, and the particle number concentration decreased when the NOx/naphthalene increased from 0.17 to 1.2. The addition of NOx in the PAM reactor favors the formation of organic nitrates through the RO_2_ + NO channel (Table S1). These organic nitrates have lower volatility that may lead to more efficient new particle formation, thus increasing the particle number concentration and decreasing the particle mode diameter. At the highest NOx/naphthalene ratio, the particle number decreased significantly although the particle mode diameter increased to 90 nm. Overall, the total particle mass decreased when the NOx/naphthalene ratio increased from 0.17 to 2.7, indicating a suppression effect of NOx on *naph*-SOA formation, which is in line with previous studies.^23,24,35^ For *naph*-SOA produced with a fixed initial N_2_O concentration (A08–A49), the particle mode diameter increased from 68 to 81 nm when the equivalent aging time increased from 0.8 to 4.9 days, while the particle number concentration kept increasing except for the experiment with the highest aging time (A49). This caused the total mass of *naph*-SOA to keep increasing with aging time. At low aging time conditions, the SOA formation was characterized by the addition of oxygenated functional groups to gas-phase molecules, which can further partition into the particle phase; therefore, the SOA yield increased with aging time. However, with further aging, cleavage of C–C bonds of intermediate products became more prominent, thus decreasing the SOA yield.^59−61^

AMS mass spectra of selected *naph*-SOA experiments are presented in Figure S3 for low and high NOx/naphthalene (0.17 vs 1.2) and aging time (0.8 vs 4.9 days) conditions. Characteristic fragments of aromatics such as *m/z* 50–51 (C_4_H_2_^+^, C_4_H_3_^+^), *m/z* 65 (C_5_H_5_^+^), and *m/z* 76–77 (C_6_H_4_^+^, C_6_H_5_^+^) were detected as the major hydrocarbon-like fragments (C_x_H_y_^+^) in all of the mass spectra. All of the mass spectra show a high intensity of *m/z* 44 (CO_2_^+^, indicative of carboxylic acids) and a low intensity of *m/z* 43 (C_2_H_3_O^+^, indicative of carbonyls). All of these features are different from typical mass spectra of SOAs from biogenic VOCs (high *m/z* 43, C_2_H_3_O^+^; *m/z* 27, C_2_H_3_^+^; *m/z* 41, C_3_H_5_^+^; *m/z* 55, C_3_H_3_O^+^).^21,36,41^ Under NOx conditions, high intensities of nitrogen-containing fragments (e.g., C_x_H_y_N_z_N^+^, NO^+^, and NO_2_^+^) are also observed, indicating intensive formation of nitro-organics. The fragment intensity ratio of NO_2_^+^/NO^+^ can be used to identify nitro-organics in the particles, as this ratio is much lower for nitro-organics (∼ 0.1) than that of inorganic nitrates (∼ 0.5, determined from NH_4_NO_3_).^62−65^ For *naph*-SOA produced with NOx, the NO_2_^+^/NO_2_^+^ ratios in the mass spectra vary between 0.09 and 0.16 (Figure S4). This ratio is very close to that of nitro-organics, further supporting the generation of nitrated *naph*-SOA in the presence of NOx.

At high NOx/naphthalene conditions, the C_x_H_y_^+^ shows a lower intensity (23% vs 26%), while the signal intensity of nitrogen-containing fragments (NO_y_^+^ and C_x_H_y_O_z_N^+^) is much higher (12% vs 7%) as compared to that of *naph*-SOA produced under a low NOx/naphthalene condition (Figure S3a). However, under a higher aging time, the intensities of CO_2_^+^ (14% vs 11%) and nitrogen-containing fragments (10% vs 8%) were much higher, while the intensities of hydrocarbon-like fragments were lower (25% vs 35%), indicating an increased carbon oxidation level and a more pronounced formation of nitrogen-containing species.

**Figure 1:**
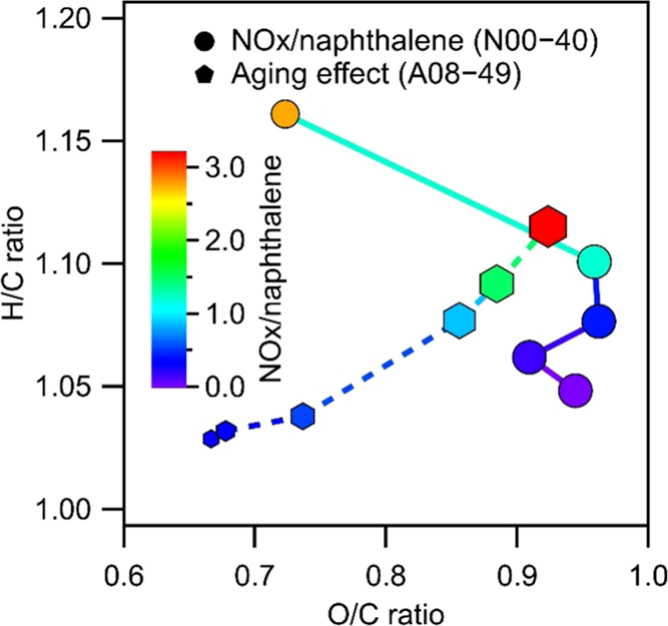
Elemental ratios (H/C vs O/C) of *naph*-SOA produced from OH oxidation. Circles show the influence of NOx/naphthalene (N00–N40), and hexagon markers exhibit the effect of aging (A08–A49) on the bulk chemical composition of *naph*-SOA. Marker colors indicate the NOx/naphthalene ratio (0–3.2), while the marker size represents the aging time (0.8–4.9 days).

The H/C and O/C ratios extracted from the AMS mass spectra data without considering the NO_y_^+^ ions are plotted in a Van Krevelen diagram (Figure 1). This plot provides an insight into the major functional groups of SOAs. For experiments conducted to test the effect of the NOx/naphthalene ratio (N00–N40), when the NOx/naphthalene ratio is below 1.2 (N00–N20), the H/C and O/C ratios do not change significantly. This is consistent with the same equivalent aging time of *naph*-SOA. When the NOx/naphthalene ratio is 2.7, the equivalent aging time of *naph*-SOA is 2.4 days. The H/C ratio is significantly higher due to a lower oxidation level of SOAs as inferred by the lower O/C. When *naph*-SOA was produced with the same initial N_2_O (A08–A49), both the H/C and O/C ratios increased with aging time (Figure 1 and S4), indicating OH addition to the aromatic rings and carboxylic acid group formation. The changes in the O/C and H/C ratios of *naph*-SOA can be well described by a linear fit. The Δ(H/C)/Δ(O/C) slope obtained in this study is 0.32 ± 0.03 (R^2^ = 0.96), indicating the addition of hydrogen atoms and the production of alcohol or peroxide. This ratio is dramatically different from that obtained for *naph*-SOA produced in the absence of NOx (slope = −0.09 ± 0.03),^36^ which implies the formation of alcohol or peroxide.^66^

### Complex Refractive Index of SOA

**Figure 2:**
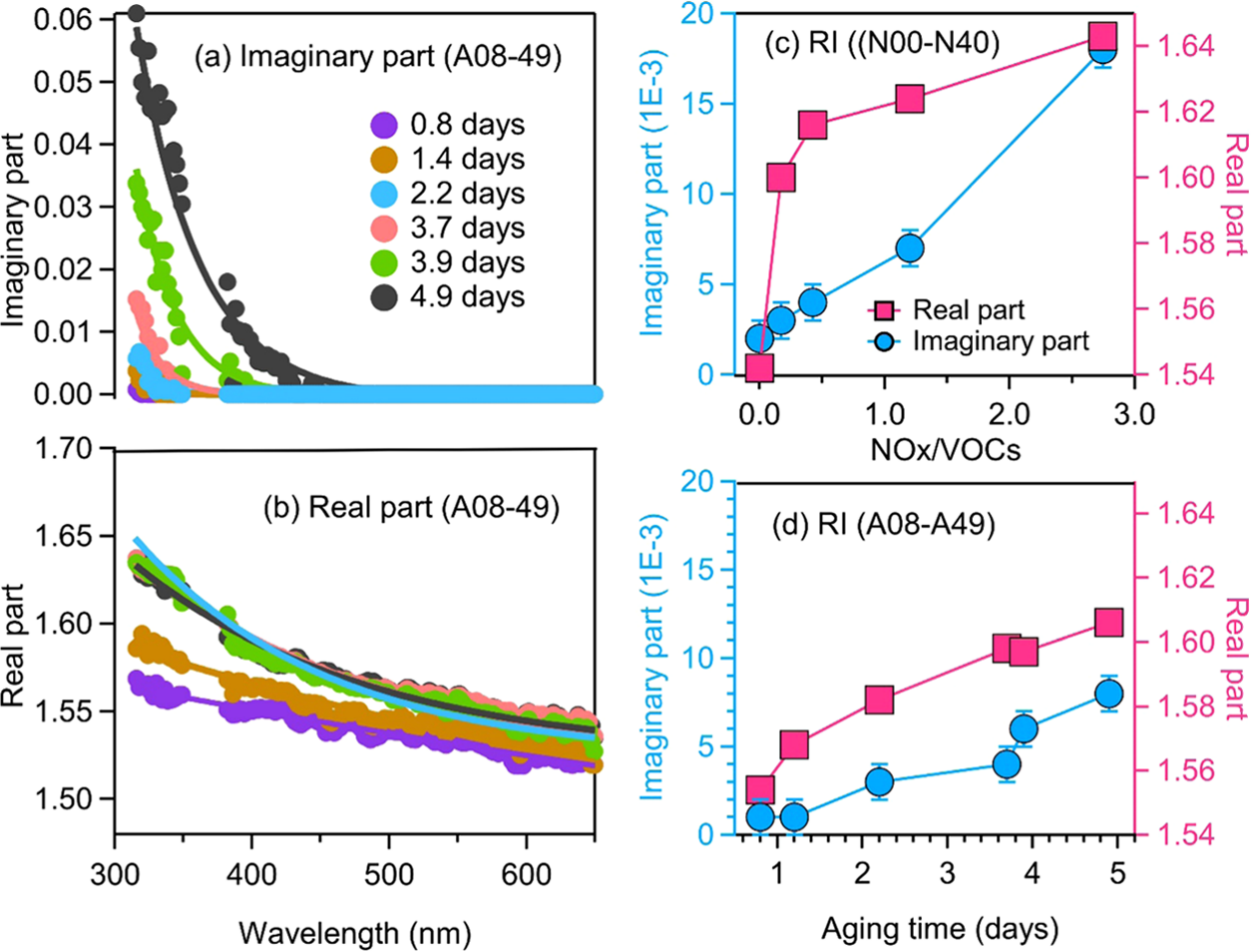
Complex refractive index (RI) of *naph*-SOA generated by OH oxidation. Panels (a, b) show BBCES-determined real and imaginary parts of RI for *naph*-SOA produced under various aging times (A08–A49). These two panels share the same bottom axis (wavelength) and legend. Panels (c, d) display the RI retrieved from PAS-CRD measurements at 404 nm for *naph*-SOA generated with different initial NOx/naphthalene ratios (c, N00–N40) and aging times (d, A08–A49), respectively. Pink squares and blue circles represent the real and imaginary parts of the RI, respectively. For BBCES, the uncertainties arise from the particle number concentration (0.3%), temperature (0.1%), pressure (0.3%), light intensity measurement (≪0.2%), and the Rayleigh scattering cross section of N_2_ (1%). The first three parameters also contribute to the uncertainties in PAS-CRD results. Moreover, uncertainty in the calibration (1.5%) was included in PAS results.

Figure 2 shows the RI of *naph*-SOA across the wavelength range of 315–650 nm as obtained by the BBCES system. This is the first wavelength-dependent RI result for *naph*-SOA in a continuous UV–vis range based on online characterization. The real part of the RI (*n*) slightly decreases with increasing wavelength, which is similar to that observed for SOA produced from biogenic and anthropogenic VOCs.^21,41,67^ For example, the *n* for *naph*-SOA produced with the aging time of 3.9 days decreased from 1.63 ± 0.02 at 315 nm to 1.53 ± 0.01 at 650 nm, with a Δ*n* of 0.1 (Figure 2b). As the aging time increases, *n* first increases until the aging time reaches 2.2 days and then is almost constant with the change in aging time. The differences are within the uncertainty of *n*. PAS-CRD measurements are more precise to differentiate small changes in the RI, and the results are shown in Figure 2c,d. There is a slight change in *n* (1.554 ± 0.001 to 1.606 ± 0.001) as the aging time increases (Figure 2d). An increase of *n* (1.542 ± 0.001 to 1.643 ± 0.001) is also observed when the NOx/naphthalene ratio increased from 0 to 2.7 (Figure 2c). A previous study by Lambe et al.^36^ reported the RI of naph-SOA from OH oxidation of naphthalene in the absence of NOx. The observed *n* values varied from 1.58 ± 0.06 to 1.66 ± 0.04 at 405 nm, comparable with our results.

The imaginary part of the RI (*k*) shows a strong spectral dependence, with *k* decreasing toward longer wavelengths (Figure 2a) as obtained from the BBCES system. For example, *k* decreases from 0.034 ± 0.004 at 315 nm to 0 at longer wavelengths. *k* increases with increasing aging time in the wavelength range where absorption is measured. *k* increased from 0.001± 0.002 to 0.061 ± 0.006 at 315 nm on increasing the aging time from 0.8 to 4.9 days (Figure 2a). This is also confirmed by the PAS measured at 404 nm, where *k* increased from 0.001± 0.001 to 0.008 ± 0.001 (Figure 2d). *k* values obtained by the PAS and BBCES agree with each other within the measurement uncertainty. The retrieved *k* values for *naph*-SOA are comparable to those measured for SOA produced from the OH oxidation of toluene and m-xylene^15^ but are substantially higher than those measured for SOA from the NO_3_/OH oxidation of biogenic VOCs.^21,68^ Without considering the aging time of SOA, results from the PAS measurements in this study are comparable to those reported for *naph*-SOA produced from OH oxidation in the absence of NOx (at 405 nm, *k* = 0.8–3.6 × 10^–3^).^36^

The mass absorption coefficient (MAC) is frequently used to describe the absorbing properties of aerosols. Figure S5 displays the MAC values obtained for *naph*-SOA produced from OH oxidation under different NOx/naphthalene ratios and aging times. Overall, the MAC values measured here have a strong spectral dependence between 300 and 500 nm, and the MAC values span a wide range under different oxidation conditions. At 405 nm, the MAC values range from 0.22 to 0.38 m^2^ g^–1^ for the samples analyzed (N00, N40, A37, A49). These values are substantially higher than those reported for *naph*-SOA produced in the absence of NOx (0.025–0.088 m^2^ g^–1^)^36^ but comparable to the value reported for *naph*-SOA generated in the presence of NOx (0.31 m^2^ g^–1^).^35^ Xie et al. also measured the MAC value for *naph*-SOA produced under the initial NOx/naphthalene ratio of 3.0.^35^ Their results at 405 nm (0.72 m^2^ g^–1^) agree well with that of *naph*-SOA produced under the NOx/naphthalene ratio of 20 by Metcalf et al. (0.81 m^2^ g^–1^)^38^ but are much higher than our measurements. This may be a result of the different oxidation conditions and experimental setups. Higher RH in this study (40% vs <15%) favors the condensation of semivolatile species that may have a lower light absorption.^69^ The XAD4-coated annular denuder upstream of the filter used by Xie et al. can also remove the semivolatile species from the particles and then increase the light absorption of the remaining less-volatile SOA. Moreover, the much higher NOx/VOC ratio (∼20) in the study by Metcalf et al.^38^ promotes the formation of stronger light-absorbing chromophores. All of these could explain the lower MAC values measured in this study as compared to the literature results.

### Formation of Chromophores in *naph*-SOA

**Figure 3:**
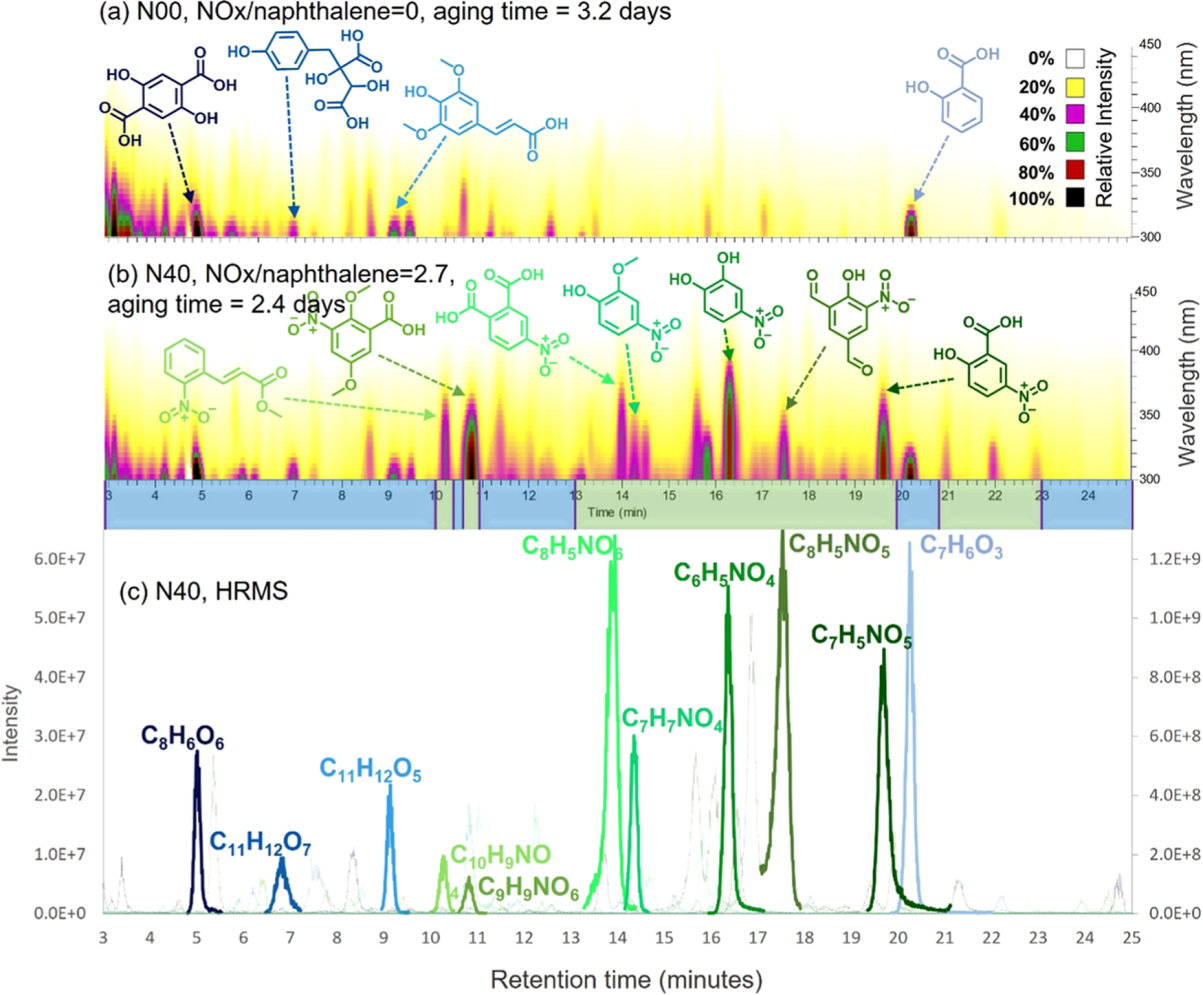
HPLC-PDA (a, b) and extracted HPLC-ESI/HRMS (c) chromatograms of the *naph*-SOA samples. Panels (a, b) are obtained for SOA generated under NOx/naphthalene ratios of 0 (N00, 3.2 days aging) and 2.7 (N40, 2.4 days aging), respectively. Possible molecular structures are proposed for these dominant absorbing species. Panel (c) shows a compilation of the selected extracted ion chromatograms (EICs) of the most abundant peaks. The color map in panels (a, b) represents the UV–vis absorbance. The left *y*-axis of panel (c) represents the intensities of C_8_H_6_O_6_, C_11_H_12_O_7_, C_11_H_12_O_5_, C_10_H_9_NO_4_, C_8_H_5_NO_6_, C_7_H_7_NO_4_, and C_8_H_5_NO_5_, while the right *y*-axis in panel (c) shows the signal intensities of C_9_H_9_NO_6_, C_6_H_5_NO_4_, C_7_H_5_NO_5_, and C_7_H_6_O_3_.

Bulk optical measurements from PAS and UV–vis absorbance have confirmed the formation of BrC chromophores in *naph*-SOA. Figure 3 shows the HPLC-PDA chromatograms (panels a and b) of *naph*-SOA generated under NOx/naphthalene ratios of 0 and 2.7 with comparable aging times (3.2 vs 2.4 days). Many strong-absorbing chromophores are observed in the HPLC elution time of 3–25 min. The chemical formulas are assigned based on accurate mass measurements, and the tentative structures are proposed based on these chemical compositions, LC retention times, UV–vis absorption spectra, and literature reports, taking into account the ring-retaining, cyclization, and ring-opening reactions of naphthalene and its oxidized intermediates.^23,25,37,39,61,70^ Oxygenated aromatic hydrocarbons (CHO, e.g., C_8_H_6_O_6_, C_6_H_6_O_3_, C_11_H_12_O_5_, C_8_H_6_O_2_, C_8_H_6_O_4_, and C_11_H_12_O_7_) are the major chromophores in *naph*-SOA produced without NOx (Figure 3a,c). This is different from the result by Xie et al., who claimed a nitroaromatic compound (C_10_H_7_NO_3_) was the major absorbing species for *naph*-SOA produced without NOx. It seems that there was a NOx contamination in Xie et al.’s chamber experiment or an artifact during the filter analysis. These CHO chromophores in *naph*-SOA produced without NOx also contribute to the light absorption of *naph*-SOA produced with NOx. However, strong light-absorbing nitroaromatics (CHON, e.g., C_6_H_5_NO_4_, C_7_H_7_NO_4_, C_7_H_5_NO_5_, C_8_H_5_NO_5_, C_8_H_5_NO_6_, C_9_H_9_NO_6_, C_10_H_9_NO_4_) are produced when NOx is involved in SOA formation (Figure 3b,c), and these species contribute significantly to the absorption of the SOA (∼ 45% of the MAC at 350–450 nm for the A49 experiment, Figure S6d). Quantitative contributions of the identified BrC chromophores to the total MAC values of the solvent-extractable BrC were calculated using the method described by Hettiyadura et al.^71^ The major absorbing CHO species, which contain hydroxy, carbonyl, and carboxylic functional groups, elute faster than the CHON species, which contain additional nitro groups, probably due to the low polarity of the −NO_2_ group, which reduces the overall polarity of CHON compounds.

Xie et al. detected eight nitroaromatic species in the SOA produced with an initial NOx/naphthalene ratio of 3.^35^ Among them, four species (C_10_H_7_NO_3_, C_10_H_7_NO_4_, C_8_H_9_NO_5_, C_6_H_5_NO_4_) contribute significantly to the light absorption at 365 (∼18%), 400 (47%), and 450 (∼18%) nm. The two most absorbing species (C_10_H_7_NO_3_ and C_10_H_7_NO_4_) found by Xie et al. are not identified as major absorbing species in our study probably due to the different RH between these two studies, which can change the gas-particle partitioning. Moreover, the application of surrogate standards by Xie et al. for quantification may also introduce large uncertainties in the determination of major chromophores. In this study, these two species elute at 28.12 and 31.26 min. Light absorption by chromophores eluting after 25 min is negligible; thus, the HPLC-PDA-HRMS analysis here is only performed within an elution time of 25 min. In contrast, C_6_H_5_NO_4_, which was found to be a minor chromophore by Xie et al., is the primary contributor to light absorption of *naph*-SOA produced in this study (Figure 3b), especially in the long-wavelength range. This is consistent with its broad UV–vis absorption spectra^55^ and higher abundance in the SOA (0.94% of the total SOA).^25^ In our study, C_6_H_5_NO_3_ and C_7_H_5_NO_5_, which elute between 19.5 and 19.8 min in our HPLC, are also detected as major products of *naph*-SOA produced under NOx conditions (1.1% and 0.5% of the total SOA) in a previous study.^25^ These two species have strong absorption in the UV–vis wavelength range and contribute significantly to the total light absorption of *naph*-SOA in this study.

### Influence of Aging Time and NO*_x_* on the Complex Refractive Index

**Figure 4:**
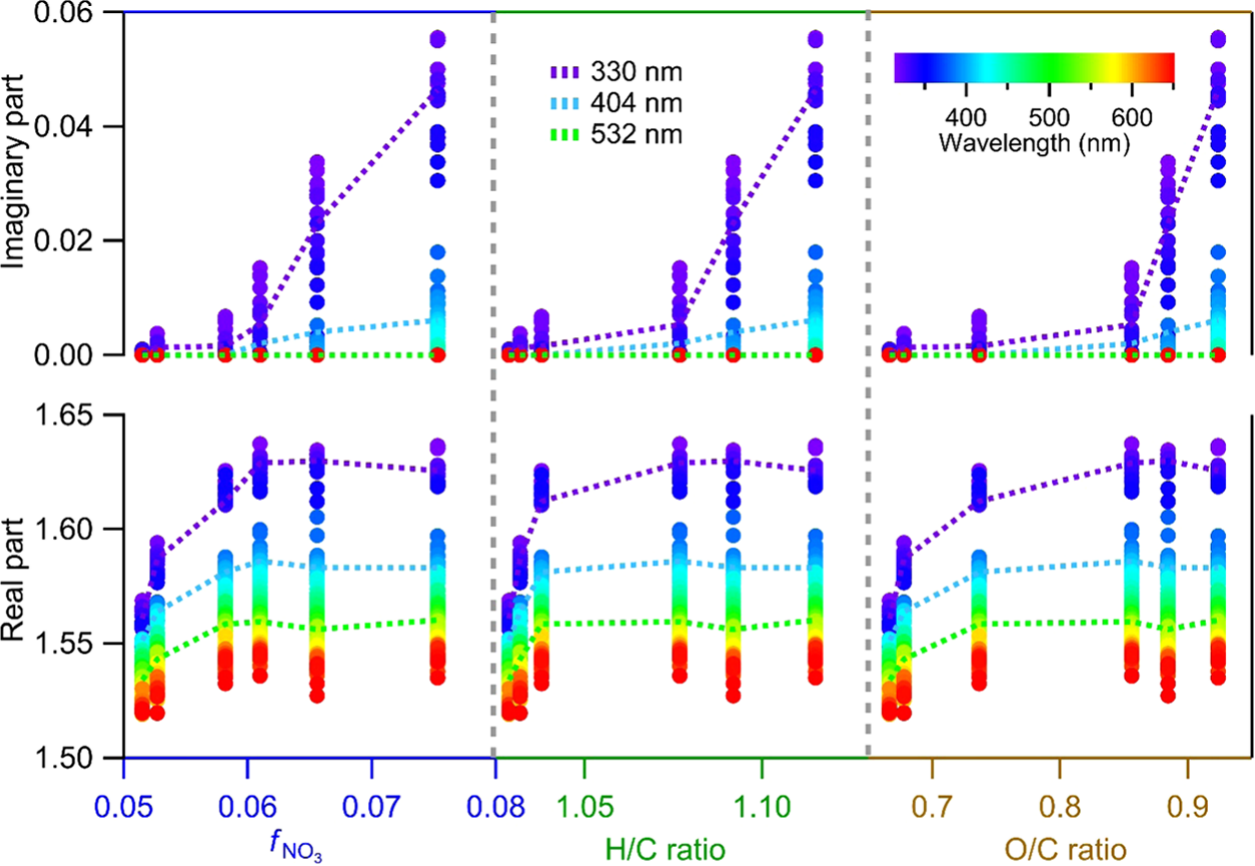
Evolution of optical properties of *naph*-SOA due to aging, as indicated by increasing H/C and O/C ratios, in the presence of NOx. The upper and lower panels show the change of the imaginary part and the real part as a function of nitrate fraction (*f*_NO_3__), H/C ratio, and O/C ratio measured with HR-ToF-AMS. The dashed lines show the RI values at 330 nm (purple), 404 nm (cyan), and 532 nm (green).

The elemental ratios (H/C, O/C) obtained from HR-ToF-AMS are commonly used to indicate the aging process. Moreover, the NO_3_ observed by the HR-ToF-AMS (inorganic nitrogen-containing ions) are fragments of nitro-organic compounds (e.g., nitroaromatics), as confirmed by the NO_2_^+^/NO^+^ ratio; thus, the nitrate fraction (*f*_NO_3__= NO_3_/(NO_3_ + organic)) can be used to indicate the fraction of nitroaromatics in *naph*-SOA. As shown in Figures 1 and S4, H/C and O/C ratios, as well as *f*_NO_3__ and NO_2_^+^/NO^+^ ratios, increase with aging time in the presence of NOx, respectively. Figure 4 plots the RI of *naph*-SOA against the *f*_NO_3__, H/C ratio, and O/C ratio. The imaginary part increases significantly with the *f*_NO_3__, H/C ratio, and O/C ratio, indicating important roles of nitro-organic compounds and aging time on the absorbing properties of *naph*-SOA. The real part of the RI increases until the aging time of *naph*-SOA reaches 3.7 days (with *f*_NO_3__ = 0.06, H/C = 1.08, and O/C = 0.86). Then, it remains almost constant as the aging time increases from 3.7 to 4.9 days.

Previous studies have reported an increase of RI when the SOA is produced from aromatic precursors in the presence of NOx than under low NOx conditions.^34,35,72,73^ The OH oxidation of naphthalene occurs through hydrogen abstraction from the aromatic ring. In the presence of NOx, this intermediate then undergoes ring-retaining reactions to produce 2-nitronaphthalene.^25,40^ In addition, cyclization and ring-opening reactions of intermediates can generate formyl-benzoic acid, terephthaladehyde, formyl-benzoic acid, 2-formyl-cinnamaldehyde, and 2-hydroxy-4-nitrobenzoic acid,^25,37,39,40,74^ which absorb light in the UV–vis wavelength range (Figure 3). Therefore, during the aging of *naph*-SOA, ring-retaining reactions introduce more oxygenated and nitro functional groups, while ring-opening reactions break the aromatic rings. In the PAM reactor, the NOx/VOC ratio increased with the aging time (Figure 1); thus, changes in the chemical composition and optical properties of *naph*-SOA are affected by both the NOx/VOC ratio and the aging time.

### Effect of NOx/Naphthalene Ratio on the RI

**Figure 5:**
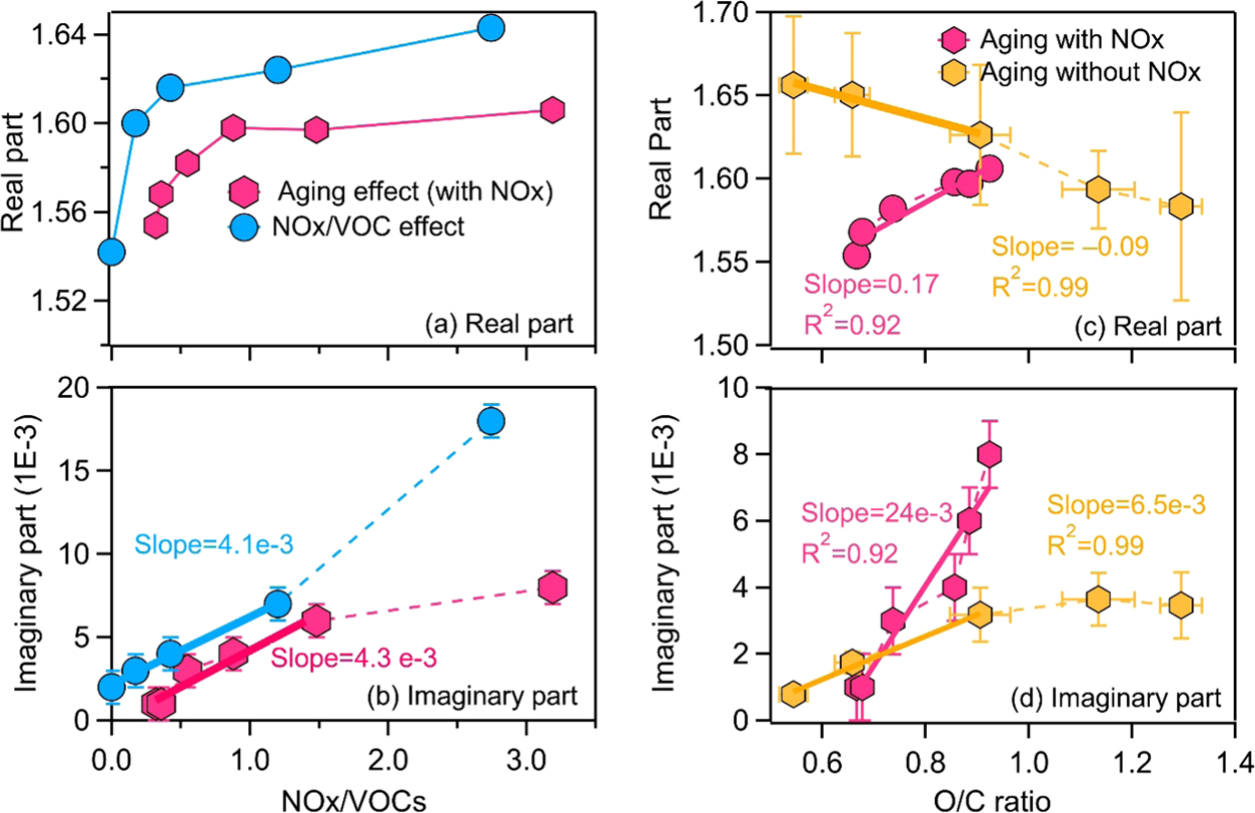
RI (404 nm) changes as a function of the NOx/VOC ratio (a, b) and aging time, which is represented by O/C (c, d). Blue markers indicate the results from experiments N00–N40, which are conducted with a fixed aging time but with different NOx/VOC ratios. Red markers indicate results from experiments A08–A49, which were performed with different aging times and NOx/VOC ratios. Brown markers represent literature results at 405 nm for *naph*-SOA produced without NOx under different aging times.^36^*k* obtained from experiment N40 was not included in the fitting (panel b) due to the shorter aging time than N00–N20. To constrain similar NOx/VOCs and O/C ratios for comparison, only part of the results is included in the fitting, as shown in panels (b)–(d).

As the aging time for generation of *naph*-SOA is fixed (N00–N40), the *f*_NO_3__ increases with increasing NOx/naphthalene ratio (Figure S4a), indicating that the reactions produce an increasing amount of CHON products (Figure S7) by adding nitro groups to the oxidized intermediates.^40^ The imaginary part of the RI for *naph*-SOA linearly increases with the NOx/naphthalene ratio with a slope of 0.004 at 404 nm (Figure 5b). As the NOx/VOC ratio increases, more RO_2_ will react with NO to produce nitro-organic compounds (Table S1) that are highly absorbing. A higher nitro-organic compound content under higher NOx/naphthalene ratios was observed by HR-ToF-AMS (Figures S3 and S4) and HPLC-HRMS (Figure S7). Siemens et al.^55^ have analyzed the double-bond equivalent value (DBE) of the assigned species. They suggest that the high DBE values of the CHON compounds retain the aromaticity and are likely nitroaromatics. These nitroaromatic compounds have an extended network of π bonds, and they possess additional oxygenated functional groups, resulting in feasible light-absorbing properties toward the longer wavelength range.^75−77^ The real part of the RI increases with increasing aging time and NOx/naphthalene ratio, especially in the lower ranges (Figure 5a). An empirical expression relates the real part of RI (*n*) positively to the molecular volume (density (ρ) divided by the molecular weight (MW)) mean polarizability (α) as follows
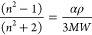
The mean polarizability can be estimated using elemental composition.^78,79^ It is positively correlated with the number of atoms in a molecule. As the NOx/VOC increases, the mean polarizability of *naph*-SOA increases and the density remains constant, while the MW decreases, as indicated by the significant decay of large molecules observed by Siemens et al.^55^ Therefore, an increase of the real part of RI is expected with the increase of the NOx/VOC ratio. At higher NOx/naphthalene ratios, the increased prevalence of NOx promotes RO_2_ to react more with NOx to form nitro-organic compounds than reacting with another RO_2_ to produce larger molecules (e.g., ROOR).

### Effect of Aging on the RI

As the aging process proceeds in the presence of NOx, more oxygenated functional groups are formed and NOx participates in the reactions, leading to the production of CHON compounds.^80^ While the functionalization can increase the light-absorbing ability of *naph*-SOA, further oxidation causes ring-opening, leading to loss of aromaticity and eventual loss of functional groups (fragmentation). Loss of aromaticity is also going to contribute to the change in RI. Therefore, the overall change of the chemical composition and optical properties of *naph*-SOA is a combined effect of functionalization and fragmentation.^81^

Figure 5 shows the RI measured by the PAS-CRD at 404 nm. As the aging time increases (indicated by the O/C ratio), both the real part and the imaginary part increase (Figure 5c,d). The *n* values show a linear dependence on the O/C ratio of *naph*-SOA, with a slope of Δ(*n*)/Δ(O/C) = 0.17 (*R*^2^ = 0.92). This is consistent with the finding for the heterogeneous OH aging of squalene and azelaic acid aerosols.^82^ These results suggest a dominant role of functionalization, leading to an increase in the mean polarizability, which results in higher *n* values. However, an opposite trend was observed by Lambe et al., who found that *n* values decrease with an increase in the O/C ratio of *naph*-SOA produced without NOx.^36^ Lambe et al. observed a significant decrease in the H/C ratio of *naph*-SOA although the O/C ratio increased with aging time, suggesting that a significant fragmentation process dominated in their experiments.

The *k* values at 404 nm linearly increase with the O/C ratio of *naph*-SOA produced under different aging times (Figure 5d). The magnitude of this increase is Δ(*k*)/Δ(O/C) = 0.024. This increase is much more significant than that observed for *naph*-SOA produced without NOx with similar O/C ratios (slope of Δ(*k*)/Δ(O/C) = 0.0065 for SOA with the O/C ratio below 1.0), indicating the dominant role of NOx in the absorption enhancement during aging. This is further confirmed by the results shown in Figure 5b. For experiments performed with varying aging times (A08–A49), the NOx/VOCs ratio also increased. The magnitude of this increase is Δ(*k*)/Δ(NOx/VOC) = 0.0043, which is similar to that of *naph*-SOA produced with the same aging time but with different NOx/VOCs ratios (N00–N40, Δ(*k*)/Δ(NOx/VOC) = 0.0041). This significant absorption enhancement is governed by a strong-absorbing CHON chromophore production under higher NOx/VOCs conditions, which promotes the RO_2_ + NO reactions (Table S1, Figures S3 and S7).

### Atmospheric Implications

The broadband refractive index across the UV–vis wavelength range was obtained for the first time for *naph*-SOA produced under atmospheric relevant conditions. The imaginary parts of RI for *naph*-SOA are comparable to those measured for SOA produced from OH oxidation of typical anthropogenic VOCs and are substantially higher than those measured for biogenic SOA. Since naphthalene is the most abundant PAH in urban atmospheres and can produce SOA with high yields, *naph*-SOA has been identified as one of the major SOA sources in urban areas by both model simulations^32^ and ambient observations.^28^ Considering the prominent absorbing properties of *naph*-SOA and its ubiquitous occurrence, we expect *naph*-SOA to be a potential BrC source in urban atmospheres and also downwind from such areas. Our lab results also provide valuable information that *naph*-SOA might help explain the previously observed prominent secondary BrC in urban areas not affected by strong biomass burning emissions.^12,83^ Therefore, *naph*-SOA should be incorporated in models to better understand the atmospheric BrC burden and its climate effects.

MAC values obtained in this study differ from those obtained for *naph*-SOA produced under dry conditions, suggesting that relevant RH conditions are critical for laboratory BrC studies. The broad range of MAC values also highlights the important role of the NOx/VOC ratio and aging time on the SOA optical properties. Chromophores typically assigned as biomass burning markers (e.g., nitroaromatics, oxygenated PAHs) were also found in *naph*-SOA, demonstrating the need for comprehensive molecular-level analysis for source apportionment and predictive understanding of BrC in the atmosphere.

We conclude that within the studied ranges, both longer aging time and a higher NOx/VOC ratio during *naph*-SOA formation yield more light-absorbing nitroaromatic species, emphasizing the influence of NOx emissions and photochemical aging on the evolution of chemical and optical properties of aerosols from real-world anthropogenic emissions. Our results indicate that elevated light absorption of the SOA downwind cities can be observed due to *naph*-SOA aging under NOx conditions during the first few days of atmospheric transport. This phenomenon was observed by Qin et al. at a suburban site downwind of Guangzhou, China, where the light absorption of fresh SOA was negligible, while aged SOA showed strong absorbing properties.^84^ Unlike previous studies that tested the NOx effect on the optical properties of SOA qualitatively using “high NOx” and “low NOx/NOx-free” conditions, this study provides a quantitative analysis of the impact of the NOx/VOC ratio on the absorption of SOA. The imaginary part of RI increases with the NOx/VOC ratio with a slope of 0.004 at 404 nm. We also quantified the aging effect and found that the *k* of naph-SOA linearly increases with the O/C ratio with a slope of 0.024. These quantitative results are useful for models to accurately capture the evolution of BrC in the ambient environment, therefore contributing to an accurate description of the potential impacts of anthropogenic emissions on the global radiative budget, air quality, and cloud feedbacks.^85^

## Supporting Information Available

Experimental setup of this study (Figure S1); particle size distribution of *naph*-SOA and the chemical composition information obtained by HR-ToF-AMS (Figures S2–S4); contributions of CHO and CHON compounds to the total cumulative intensities and the MAC (Figures S5–S7); contribution of OH, O_3_, and NO_3_ in the naphthalene oxidation (Table S1); and the refractive index of various types of SOA at 404 nm (Table S2) (PDF)
