# Supplementary material for: Optical Properties of Secondary Organic Aerosol Produced by Photooxidation of Naphthalene under NOx Condition
Source: Environ Sci Technol. 2022 Apr 6;56(8):4816–27. doi: 10.1021/acs.est.1c07328 (PMC9022426; doi:10.1021/acs.est.1c07328)
Supplement: Supplementary file 1 — es1c07328_si_001.pdf [file es1c07328_si_001.pdf]

Optical Properties of Secondary Organic Aerosol Produced by Photooxidation of Naphthalene under NOx Condition

Quanfu He,[†] Chunlin Li,[†] Kyla Siemens,[§] Ana C. Morales,[§] Anusha Priyadarshani Silva Hettiyadura,[§] Alexander Laskin,[§,▿] and Yinon Rudich[†,*]

[†] Department of Earth and Planetary Sciences, Weizmann Institute of Science, Rehovot 76100, Israel

[§] Department of Chemistry, [▿] Department of Earth, Atmospheric, and Planetary Sciences, Purdue University, West Lafayette, IN 47907, USA

Correspondence to: Yinon Rudich (yinon.rudich@weizmann.ac.il)

This supporting information file has 10 pages, including

2 text: Text S1-2

2 table: Table S1-2

7 figures: Figure S1-S7

14 references

Supporting Methods (Text S1-2)

Text S1. Estimation of the contributions of OH, O_3, and NO_3 in the naphthalene consumption.
Text S2. Chemical composition measurement by HR-ToF-AMS.

Supporting Figures (Figures S1-S7)

Figure S1. Experimental setup

Figure S2. Particle size distribution of *naph*-SOA produced by OH oxidation.

Figure S3. Fragment composition of the *naph*-SOA measured by HR-ToF-AMS.

Figure S4. Bulk chemical composition of the *naph*-SOA measured by HR-ToF-AMS.

Figure S5. The Mass Absorption Coefficient (MAC) of *naph*-SOA produced by OH oxidation.

Figure S6. The contributions of oxygenated compounds (CHO), nitroaromatics (CHON), and unretained (unretained) species to the MAC. The upper two panels (a and b) show the

Figure S7. Chemical composition of *naph*-SOA obtained by ESI-HRMS.

Supporting Tables (Tables S1-2)

Table S1. The contribution of OH, O_3, and NO_3 in the naphthalene oxidation.
Table S2. Refractice index of various types of SOA at 404 nm.

Supporting Methods

Text S1. Estimation of the contributions of OH, O_3, and NO_3 in the naphthalene consumption.

We used the Aerodyne photochemical model implemented in MATLAB (Mathworks) to simulate the reactions in the PAM reactor.[1] The concentrations of radicals and oxidant species generated in the reactor were then calculated. Model input parameters included the temperature, pressure, [O_3], [H_2O], [N_2O], the mixing ratio of naphthalene, and the mean residence time. The OH exposures were determined by tracking the SO_2 decay in the PAM reactor. Photon flux is essential to run the photochemical model. However, it is hard to accurately measure the photon flux inside the PAM reactor. Therefore, we determined this parameter by varying the model input photon flux value until the model output OH exposure matches that measured by the SO_2 method. An integrated time step of 5 ms was used for the differential equations which describe the chemistry. By considering the reaction rate constants of naphthalene with OH radical (2.63×10^{-11} cm^3 $molecule^{-1}$ s^{-1}),[2] O_3 (3×10^{-19} cm^3 $molecule^{-1}$ s^{-1}),[3] and NO_3 (3.65×10^{-28} cm^6 $molecule^{-2}$ s^{-1}),[4] we estimated the contributions of these oxidants in the consumption of naphthalene. Table S2 summarised the OH, O_3, NO_2, and NO_3 exposures inside the PAM reactor. The fractions of naphthalene consumed by the OH, O_3, and NO_3 were also listed. The simplified RO_2 chemistry is also included in this model. Thus, the relative importance of NO channel (RO_2+NO) and HO_2 channel (RO_2+HO_2) in the chemical fate of RO_2 were estimated.

Text S2. Chemical composition measurement by HR-Tof-AMS

Non-refractory components, e.g., organics and nitrate, were measured by a high-resolution time-of-flight aerosol mass spectrometer (HR-ToF-AMS, Aerodyne Research Inc., Billerica, MA, USA). The HR-ToF-AMS was operated by alternating between the high-sensitivity V mode and the high-resolution W mode to measure the chemical compositions of SOA. The toolkit Pika 1.24 was used for analyzing the chemical compositions of the SOA.[5,6] W mode data was used to extract the fragments composition and elemental ratios of *naph*-SOA. The ions O^+, OH^+, H_2O^+, and CO^+ were included in the $CxHyO_2^+$ group because concentrations of these species were calculated from the organic CO_2^+ ion abundance.[7] Correction for the elemental ratios was achieved by applying the Improved-Ambient method.[6] The ionization efficiency of the AMS was calibrated using 350 nm ammonium nitrate particles. The PTof was calibrated using size selected ammonium nitrate particles with diameters in the range of 40 to 900 nm. Particle-free air, which was obtained by passing the air flow from the PAM reactor through a HEPA filter, was measured for at least 50 min before particle composition measurement to determine the background signals from major gases.

Supporting Figures

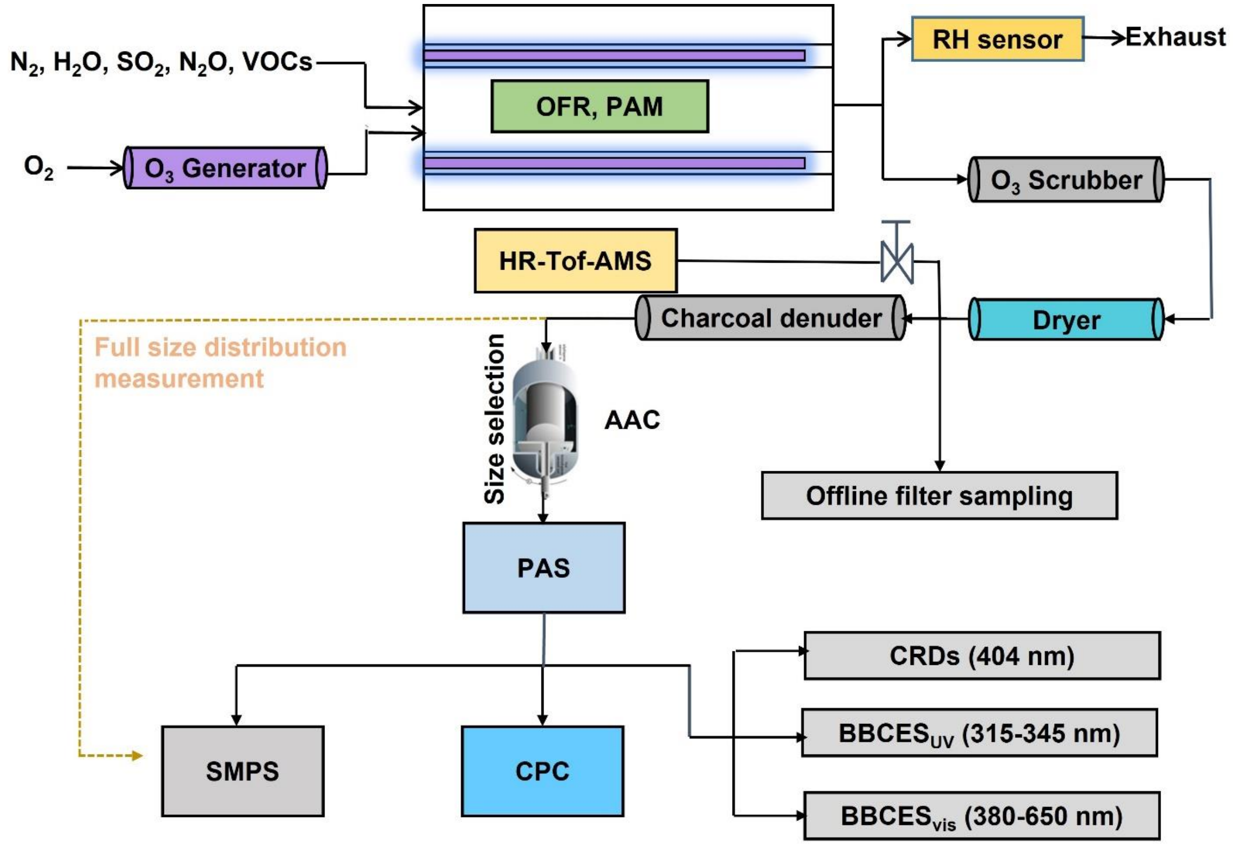

Figure S1. Experimental setup. *Naph*-SOA is produced in a potential aerosol mass oxidation flow reactor (PAM-OFR). The particles are dried by silica gel denuders and the O_3 is removed before entering the instruments. Chemical composition of the generated *naph*-SOA is characterized by a high-resolution time-of-flight mass spectrometer (HR-ToF-AMS) and the particle size is measured by a scanning mobility particle sizer (SMPS). The particles are size-selected by an aerodynamic particle classifier (AAC, Cambustion UK). The total number concentration and size distribution of the selected particles are measured by a condensation particle counter (CPC) and a scanning mobility particle sizer (SMPS). Meanwhile, the absorption and extinction of the selected particles are measured using a photoacoustic absorption spectrometer (PAS, 404 nm), cavity ring down spectrometer (CRDS, 404 nm), and a broadband cavity enhanced spectrometer which consists of a UV channel ($BBCES_{UV}$, 315−345 nm) and a visible channel ($BBCES_{Vis}$, 380−650 nm). Filter samples are collected for offline HPLC-PDA-ESI/HRMS analysis.

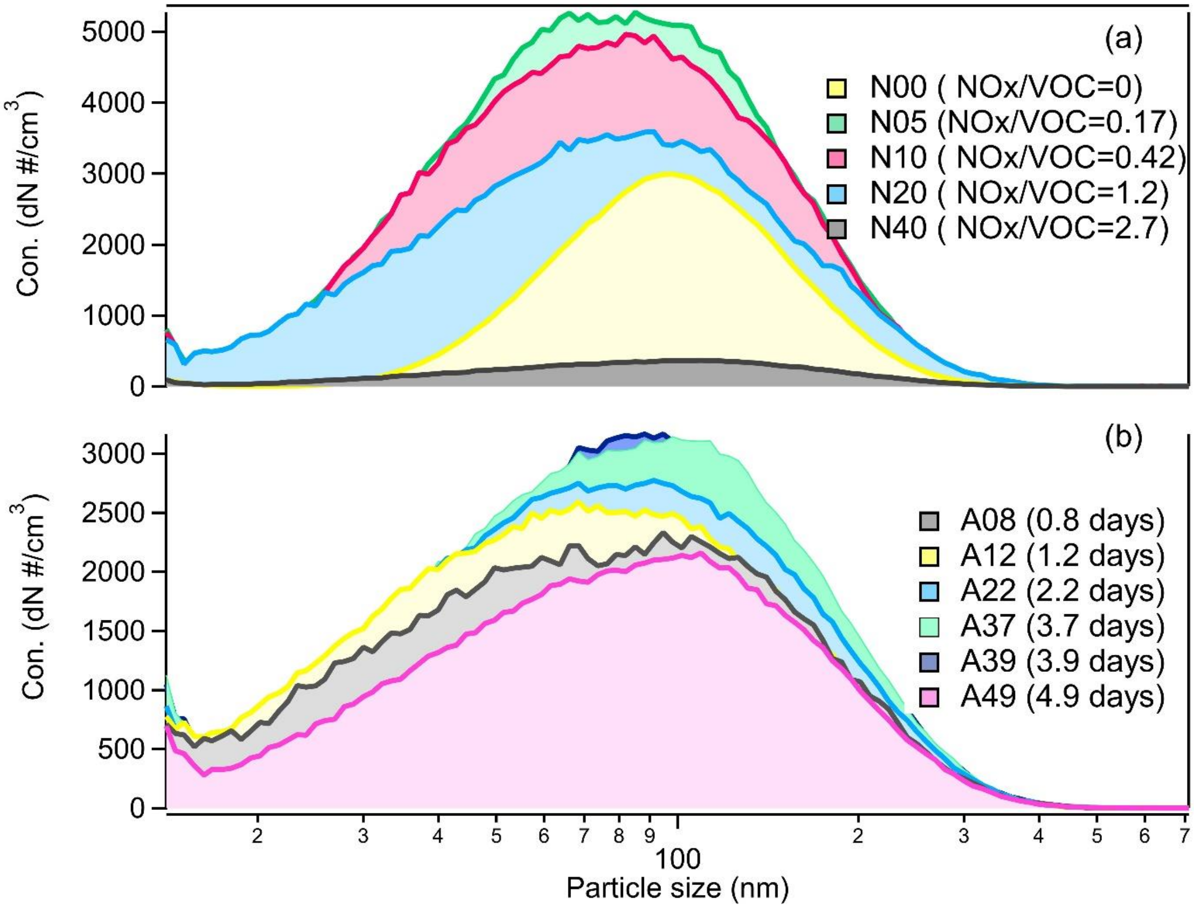

Figure S2. Particle size distribution of *naph*-SOA produced by OH oxidation. Panel (a) shows the results with varying initial N_2O while panel (b) presents the results with different aging time.

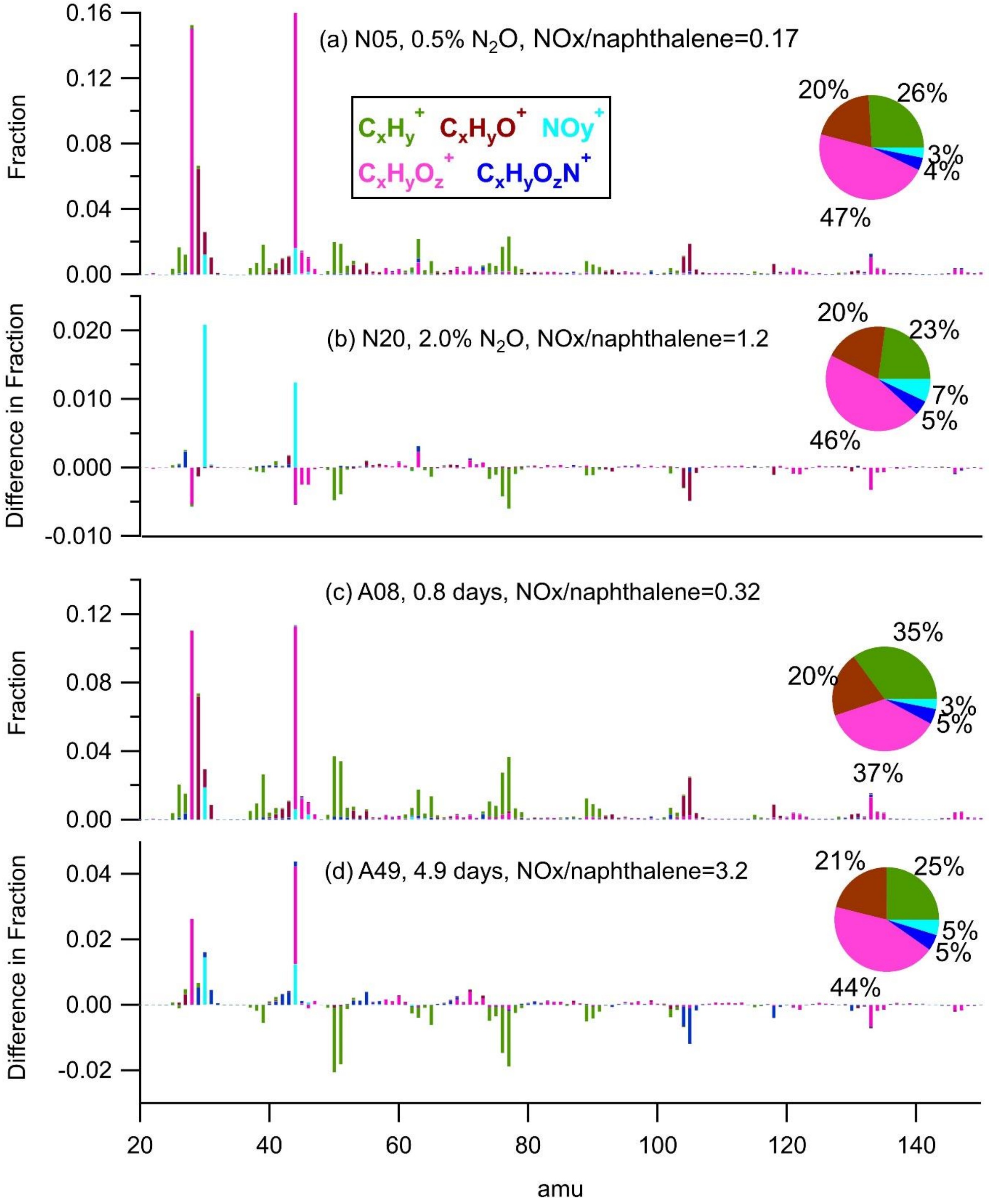

Figure S3. Fragment composition of the *naph*-SOA measured by HR-ToF-AMS. The pie charts show the relative fragment composition, including $C_xH_y^+$, $C_xH_yO^+$, $C_xH_yO_z^+$, $C_xH_yO_iN_j^+$, and NO_y^+, (x, y, z, j ≥ 1, i≥ 0). Ions of $C_xH_yN^+$, $C_xH_yON^+$, $C_xH_yO_zN^+$ in the mass spectra are categorized to $C_xH_yO_iN_j^+$. Panel (a) and (c) are the mass spectra of *naph*-SOA while panels (b) and (d) are the differences in the mass spectra as compared to that in panels (a) and (c) respectively.

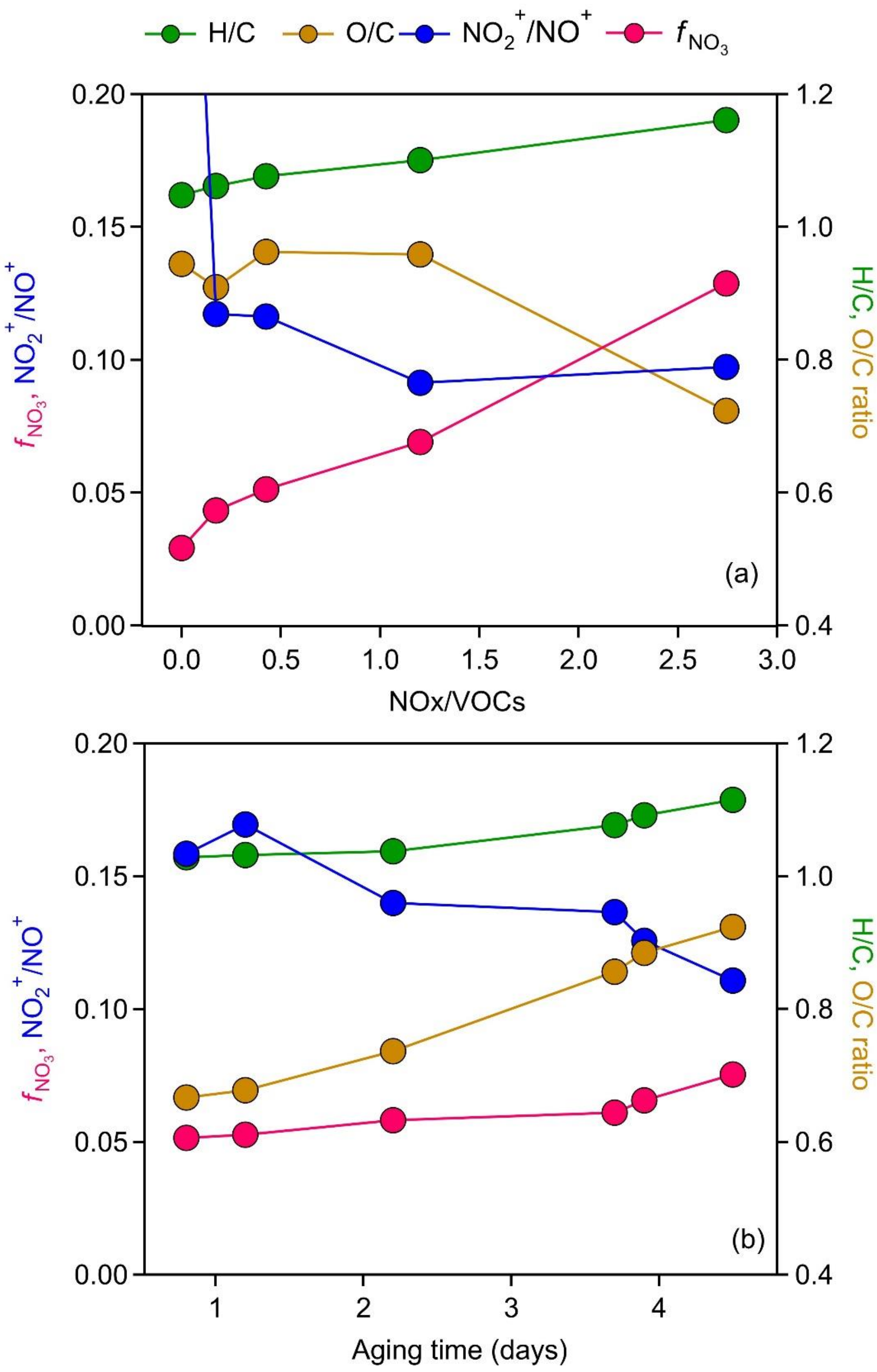

Figure S4. Bulk chemical composition of *naph*-SOA measured by HR-ToF-AMS. Panels (a) and (b) show the effect of NOx/naphthalene (N00-N40) and aging time (A08-A49) on the chemical composition of *naph*-SOA, respectively.

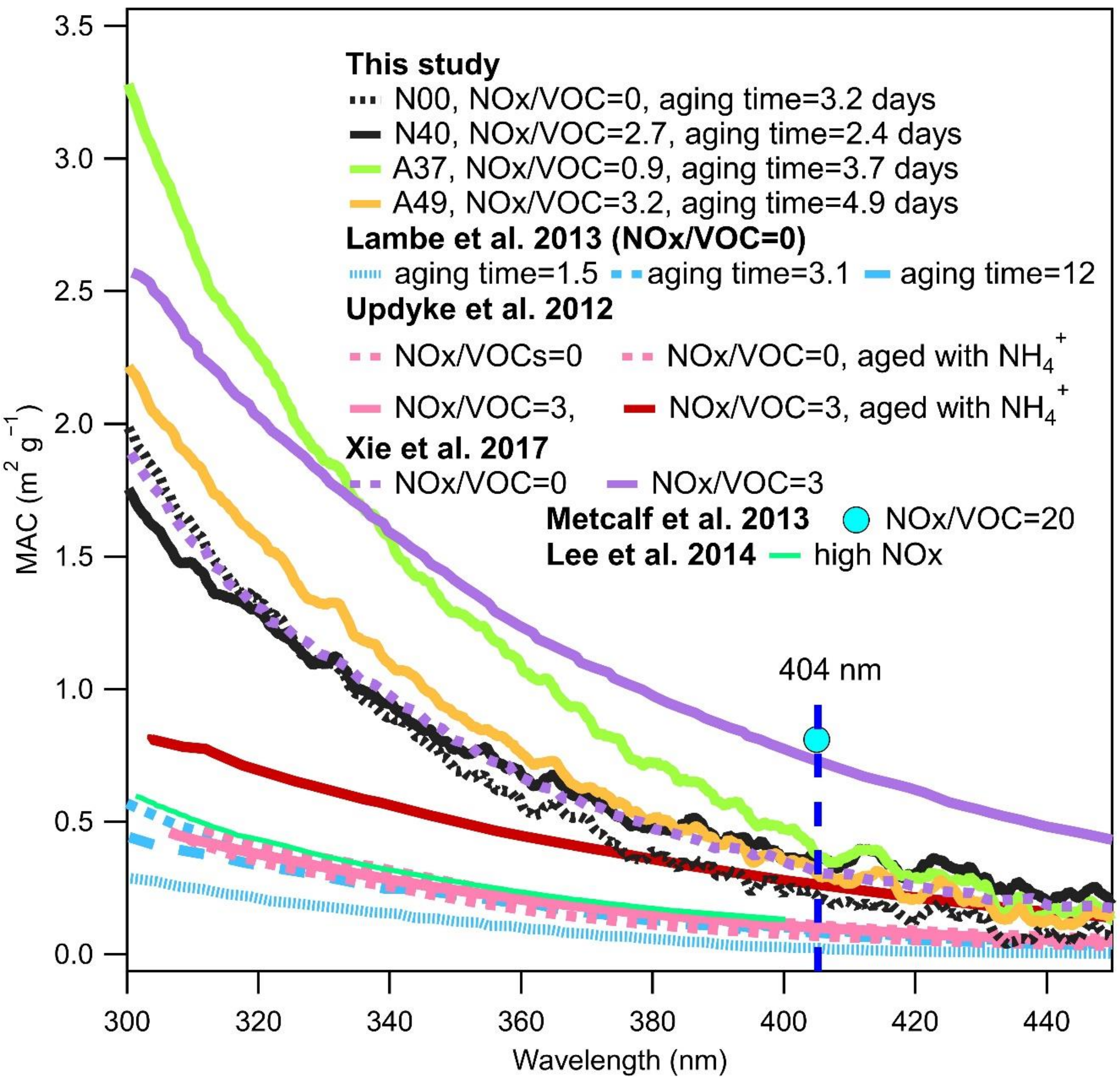

Figure S5. The Mass Absorption Coefficient (MAC) of *naph*-SOA produced by OH oxidation. The MAC results were acquired by UV-vis measurements of acetonitrile extracted *naph*-SOA.[7-10] Data from Metcalf et al. is calculated from PAS measurement.[11] Dashed lines indicate data obtained for *naph*-SOA produced under NOx-free conditions, while solid lines represent *naph*-SOA produced under NOx conditions. The blue dashed line indicates wavelength 404 nm.

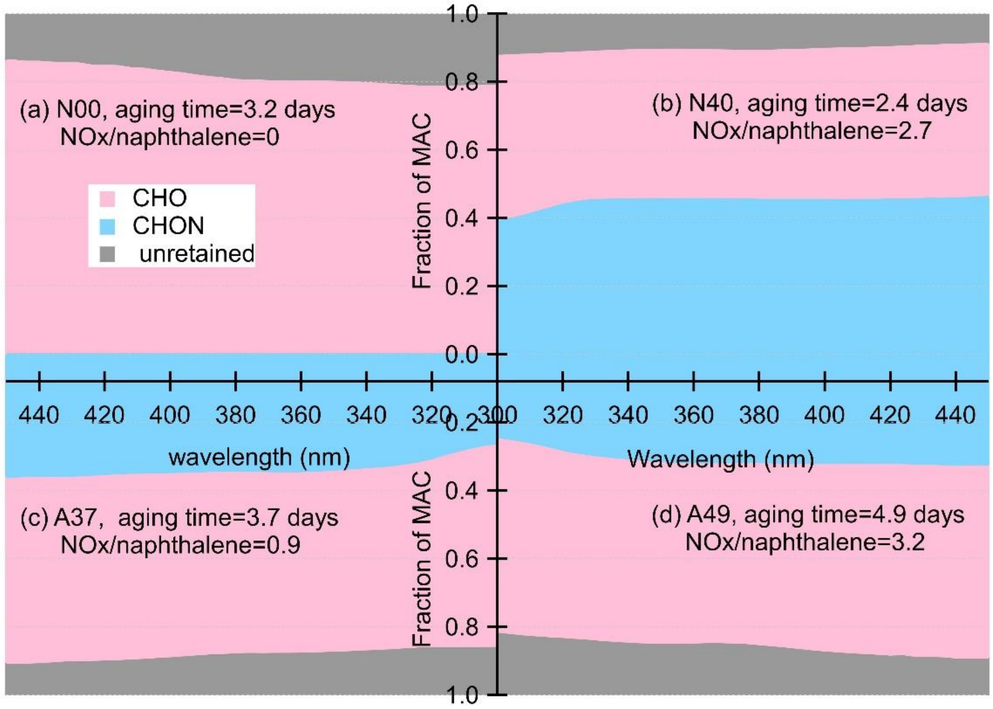

Figure S6. The contributions of oxygenated compounds (CHO), nitroaromatics (CHON), and unretained (unretained) species to the MAC. The upper two panels (a and b) show the influence of NOx/naphthalene ratio while the lower two panels (c and d) present the effect of aging time.

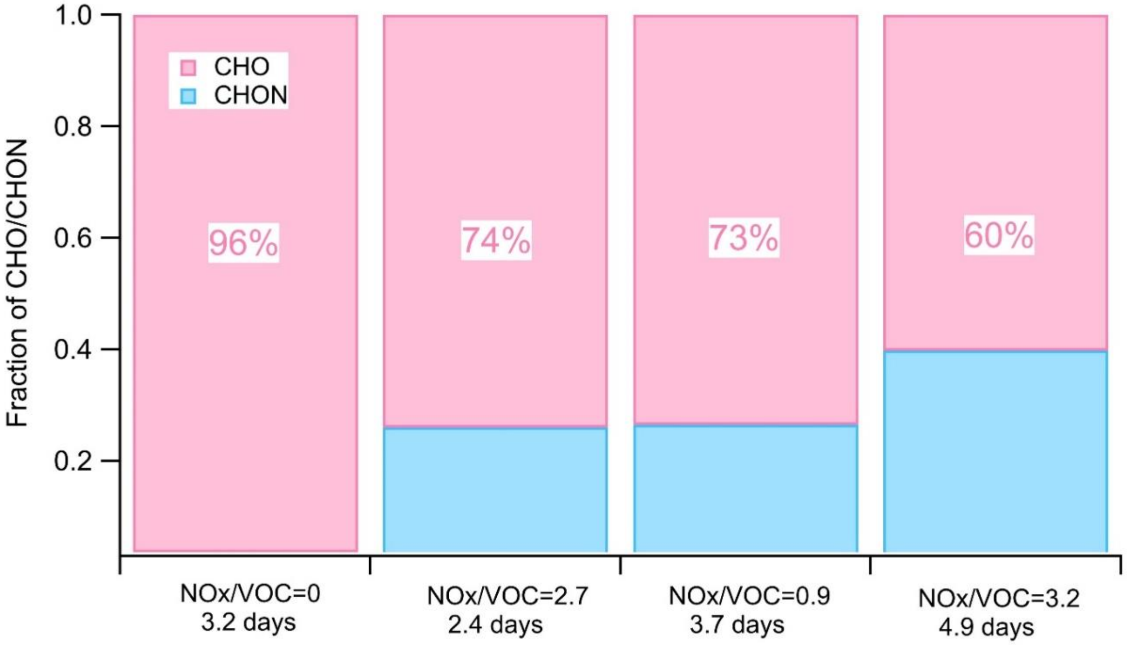

Figure S7. Chemical composition of *naph*-SOA obtained by HPLC-ESI-HRMS. The contributions of cumulative intensities of CHO (pink) and CHON (light blue) components over HPLC retention time of 3-25 min for *naph*-SOA produced with different NOx/naphthalene ratio and aging time.

Table S1. The contribution of OH, O_3, and NO_3 in the naphthalene oxidation.

Experiment ID	Naphthalene ppbv	NOx ppbv	OH exposure molecules cm^{-3} s	O_3 exposure molecules cm^{-3} s	NO_3 exposure molecules cm^{-3} s	NO_2 exposure molecules cm^{-3} s	RO_2+NO /RO_2+HO_2	Naphthalene consumption OH	O_3	NO_3
A08	345	109	1.04E+11	2.07E+17	6.23E+12	9.18E+13	0.02	0.977	0.022	0.000
A14	345	124	1.81E+11	2.04E+17	1.09E+13	2.58E+14	0.05	0.986	0.013	0.001
A22	345	188	2.85E+11	1.93E+17	1.38E+13	6.04E+14	0.17	0.990	0.008	0.002
A37	345	303	4.80E+11	1.42E+17	1.42E+13	1.61E+15	0.70	0.993	0.003	0.004
A39	345	511	5.05E+11	1.08E+17	1.41E+13	1.76E+15	0.80	0.994	0.002	0.004
A49	345	1111	6.35E+11	5.35E+16	1.30E+13	2.70E+15	1.57	0.995	0.001	0.004
N00	493	0	4.15E+11	1.43E+17				0.996	0.004	0.000
N05	493	85	4.15E+11	1.35E+17	7.10E+12	1.85E+14	0.04	0.996	0.004	0.000
N10	493	209	4.15E+11	1.28E+17	1.01E+13	5.35E+14	0.02	0.996	0.003	0.001
N20	493	592	4.15E+11	9.28E+16	1.01E+13	2.12E+15	1.35	0.994	0.003	0.004
N40	493	1352	3.11E+11	5.17E+16	5.67E+12	7.35E+15	10.9	0.988	0.002	0.010

Table S2. The refractive index of various types of SOA at 404 nm.

SOA types	Instrument	Real part	Imaginary part	Wavelength (nm)	Reference
Naphthalene+OH+NOx	PAS, CRD	1.554(±0.001) −1.643(±0.002)	0.001(±0.001) −0.018(±0.001)	404	This study
Naphthalene+OH	PAS, CRD	1.58((±0.0.04) −1.66(±0.0.06)	0.0008−0.0036	405	[7]
Naphthalene+OH+NOx	UV/vis		0.03±0.0028	400	[9]
Naphthalene+OH	UV/vis		0.013±0.0011	400	[9]
Toluene+OH+NOx	Elipsometer	1.546(±0.004) −1.571(±0.005)	0.0017(±0.0002) −0.015(±0.002)	405	[12]
m-xylene+OH+NOx	Elipsometer	1.531(±0.006) −1.565(±0.007)	0.0008(±0.0001) −0.003(±0.0003)	405	[12]
BVOCs+NO_3	CRD	1.439(±0.001) −1.579(±0.001)		404	[13]
α-pinene+O_3	Elipsometer	1.484		404	[14]
limonene+O_3	Elipsometer	1.489		404	[14]

References

(1) Lambe, A. T.; Massoli, P.; Zhang, X.; Canagaratna, M.; Nowak, J.; Yan, C.; Nie, W.; Onasch, T.; Jayne, J.; Kolb, C.; Davidovits, P.; Worsnop, D.; Brune, W., Controlled nitric oxide production via O(1D) + N2O reactions for use in oxidation flow reactor studies. *Atmos. Meas. Tech.* **2017,** *10*, (6), 2283-2298.

(2) Roueintan, M. M.; Cho, J.; Li, Z., Kinetics Investigation of Reaction of Naphthalene with OH Radicals at 1–3 Torr and 240–340 K. *Int J Chem Kinet* **2014,** *46*, (10), 578-586.

(3) Atkinson, R.; Aschmann, S. M., Kinetics of the reactions of naphthalene, 2-methylnaphthalene, and 2,3-dimethylnaphthalene with OH radicals and with O3 at 295 ± 1 K. *Int J Chem Kinet* **1986,** *18*, (5), 569-573.

(4) Phousongphouang, P. T.; Arey, J., Rate Constants for the Gas-Phase Reactions of a Series of Alkylnaphthalenes with the Nitrate Radical. *Environ. Sci. Technol.* **2003,** *37*, (2), 308-313.

(5) Aiken, A. C.; DeCarlo, P. F.; Jimenez, J. L., Elemental analysis of organic species with electron ionization high-resolution mass spectrometry. *Anal. Chem.* **2007,** *79*, (21), 8350-8358.

(6) Canagaratna, M. R.; Jimenez, J. L.; Kroll, J. H.; Chen, Q.; Kessler, S. H.; Massoli, P.; Ruiz, L. H.; Fortner, E.; Williams, L. R.; Wilson, K. R.; Surratt, J. D.; Donahue, N. M.; Jayne, J. T.; Worsnop, D. R., Elemental ratio measurements of organic compounds using aerosol mass spectrometry: characterization, improved calibration, and implications. *Atmos. Chem. Phys.* **2015,** *15*, (1), 253-272.

(7) Lambe, A. T.; Cappa, C. D.; Massoli, P.; Onasch, T. B.; Forestieri, S. D.; Martin, A. T.; Cummings, M. J.; Croasdale, D. R.; Brune, W. H.; Worsnop, D. R.; Davidovits, P., Relationship between Oxidation Level and Optical Properties of Secondary Organic Aerosol. *Environ. Sci. Technol.* **2013,** *47*, (12), 6349-6357.

(8) Updyke, K. M.; Nguyen, T. B.; Nizkorodov, S. A., Formation of brown carbon via reactions of ammonia with secondary organic aerosols from biogenic and anthropogenic precursors. *Atmos. Environ.* **2012,** *63*, 22-31.

(9) Xie, M.; Chen, X.; Hays, M. D.; Lewandowski, M.; Offenberg, J.; Kleindienst, T. E.; Holder, A. L., Light Absorption of Secondary Organic Aerosol: Composition and Contribution of Nitroaromatic Compounds. *Environ. Sci. Technol.* **2017,** *51*, (20), 11607-11616.

(10) Lee, H. J.; Aiona, P. K.; Laskin, A.; Laskin, J.; Nizkorodov, S. A., Effect of Solar Radiation on the Optical Properties and Molecular Composition of Laboratory Proxies of Atmospheric Brown Carbon. *Environ. Sci. Technol.* **2014,** *48*, (17), 10217-10226.

(11) Metcalf, A. R.; Loza, C. L.; Coggon, M. M.; Craven, J. S.; Jonsson, H. H.; Flagan, R. C.; Seinfeld, J. H., Secondary Organic Aerosol Coating Formation and Evaporation: Chamber Studies Using Black Carbon Seed Aerosol and the Single-Particle Soot Photometer. *Aerosol Sci. Tech.* **2013,** *47*, (3), 326-347.

(12) Liu, P. F.; Abdelmalki, N.; Hung, H. M.; Wang, Y.; Brune, W. H.; Martin, S. T., Ultraviolet and visible complex refractive indices of secondary organic material produced by photooxidation of the aromatic compounds toluene and m-xylene. *Atmos. Chem. Phys.* **2015,** *15*, (3), 1435-1446.

(13) He, Q.; Tomaz, S.; Li, C.; Zhu, M.; Meidan, D.; Riva, M.; Laskin, A.; Brown, S. S.; George, C.; Wang, X.; Rudich, Y., Optical Properties of Secondary Organic Aerosol Produced by Nitrate Radical Oxidation of Biogenic Volatile Organic Compounds. *Environ. Sci. Technol.* **2021,** *55*, (5), 2878-2889.

(14) Liu, P. F.; Zhang, Y.; Martin, S. T., Complex Refractive Indices of Thin Films of Secondary Organic Materials by Spectroscopic Ellipsometry from 220 to 1200 nm. *Environ. Sci. Technol.* **2013,** *47*, (23), 13594-13601.
